# Estimating the information value of polymorphic sites using pooled sequences

**DOI:** 10.1186/1471-2164-15-S6-S20

**Published:** 2014-10-17

**Authors:** Ketil Malde

**Affiliations:** 1 Institute of Marine Research, Nordnesgaten 50, Bergen, Norway

**Keywords:** Population genetics, SNP identification, information theory, diagnostic SNPs, expected site information

## Abstract

**Background:**

High-throughput sequencing is a cost effective method for identifying genetic variation, and it is currently in use on a large scale across the field of biology, including ecology and population genetics. Correctly identifying variable sites and allele frequencies from sequencing data remains challenging, in large part due to artifacts and biases inherent in the sequencing process. Selecting variants that are diagnostic is commonly done using diversity statistics like *F_ST_*, but these measures are not ideal for the task.

**Results:**

Here, we develop a method that directly calculates the expected amount of information gained from observing each variant site. We then develop and implement a conservative estimator that takes into account uncertainity introduced by sampling bias and sequencing error. This estimator is applied to simulated and real sequencing data, and we discuss how it performs compared to the commonly used existing methods for identifying diagnostic polymorphisms.

**Conclusion:**

The expected information content gives an easy to interpret measure for the usefulness of variant sites. The results show that we achieve a clear separation between true variants and noise, allowing us to select candidate sites with a high degree of confidence.

## Background

### SNP Selection

A large part of the genetic variation in a species come in the form of single nucleotide polymorphisms (SNPs) [1]. Technological advances in high-throughput sequencing have made it possible to detect variations on genome-wide scales, also for non-model species. With current developments in high resolution genotyping technologies like SNP arrays and high-throughput mass spectrometry, SNP analysis is quickly becoming an indispensable tool in many fields of biology.

In spite of improvements to technology, SNP analysis is still limited by genotyping cost and capacity. It therefore remains an important challenge to find a set of SNP markers that is as effective and efficient as possible. To be precise, we want to identify the minimal set of SNPs that must be examined in order to draw conclusions with an acceptable certainty - viz., the SNPs that are most *informative *for the task at hand. For instance, when selecting SNPs that are diagnostic (i.e., that can be used to identify individuals as belonging to one of two or more groups), we would like to pick a small set of sites that provide the most information about the individual's group affiliation. Although one could achieve the same certainty (at a somewhat higher cost) using a larger set of individually less informative SNPs, this would also increase the risk of *overfitting *the model to the data. Careful selection of SNPs is therefore not just an issue of economy and expedience, but also of accuracy.

### Diagnostic SNP identification

In practice, diagnostic SNPs are usually identified and ranked or selected using some variation of the following procedure:

First, samples are collected from individuals from the populations of interest, and DNA is extracted and sequenced to a depth deemed reasonable in terms of cost and benefit. Sequencing each sample individually is advantageous for reliably detecting rare alleles and to ensure a more complete SNP discovery [2], and is less sensitive to variation in molarity in the samples [3,2]. In spite of these advantages, collecting multiple samples in pools before sequencing can still be more cost effective, in particular for novel SNP discovery in less well-studied species and when sample material is abundant [4]. The sequence data is typically filtered for quality and contamination, mapped to a reference genome sequence using a short read alignment program, and putative SNPs are identified when reads differ from each other or from the reference.

The set of putative SNPs are then evaluated using some diversity statistic (e.g., *F_ST_*), or statistical confidence in allele frequency difference (e.g., using Fisher's exact test, [5]). Often several measures are used, and candidates are typically filtered on one criterion (e.g., *p*-values), and then ranked using the other (e.g., *F_ST_*). Sites can also be excluded based on coverage and more specific error estimates using base quality or mapping quality.

In practice, some additional care is often taken in the selection of candidate sites. For instance, one might require a certain minimum distance between sites in the genome in order to avoid unwanted correlations, or exclude sites in regions with low average mapping quality.

### Challenges with this approach

There are many statistics that could be used to identify diagnostic SNPs (the properties of several such statistics are reviewed by Rosenberg [6], other options are discussed by Zhou et al. [7]), but *F_ST _*is perhaps most commonly used [8], and is readily calculated from identified allele frequencies.

Unfortunately, *F_ST _*is less than ideal for several reasons. It is a population genetics statistic, and must be calculated using some estimator. There exist several different options (e.g., reviews by Weir and Hill [9] and Holsinger and Weir [10], others are suggested by Karlsson et al. [11] and Fumagalli et al. [8]) which can give different results, and thus *F_ST _*statistics may not be directly comparable between studies. *F_ST _*is not robust to errors in the data, something that becomes a challenge with the relatively high number of errors and large number of candidate sites that typically arise from sequencing data. When coverage is low, a low number of sequencing errors can shift statistics substantially, and the highest *F_ST _*scores tend to come from sites with low coverage. To counter this, coverage thresholds can be used, but this excludes a substantial fraction of candidate sites. And, although commonly used in this role, *F_ST _*is controversial as a measure of differentiation. In particular, where heterozygosity *within *populations is high, *F_ST _*will be lowered, regardless of the differences *between *populations [12].

An alternative (or complimentary) approach is to use *p*-values for allele difference, usually calculated using Fisher's exact test, but other options are also possible [7]. One challenge here is that although we are usually interested in the *magnitude *of the allele differences, this is only taken indirectly into account by *p*-values. Variation in sequencing coverage means that sites with high coverage will tend to have higher confidence, even if the actual allele difference is small [13]. Even with no difference between population, sampling will introduce artificial differences, which will result in significant *p*-values if the coverage is sufficiently high. In addition, Fisher's exact test does not generally take into account the possibility of errors - the observation of a single allele will exclude an underlying frequency of zero for the observed allele, even if that may well be the case if the observation is an error.

### Outline

In the following, we derive a method for calculating the expected information to be gained from genotyping a specific site, and argue that this is a more intuitive and useful measure for evaluating diagnostic SNPs than the commonly used alternatives. We will first describe how to calculate the expected informatino given *a priori *knowledge of allele frequency, we will then proceed to develop a method to make a conservative estimate for this statistic, taking into account sampling bias and uncertainty in the data. Finally, we provide an implementation, and discuss the results from applying it to both simulated and real data sets.

## Methods

### Expected site information

Given the drawbacks to using *F_ST _*discussed above, it is perhaps tempting to instead use some other measures, like nucleotide diversity or absolute difference in allele frequency. However, it is easy to see that nucleotide diversity per site (defined as the probability of samples having different alleles, i.e., *p*(1 *− q*) + (1 *− p*)*q *where *p *and *q *are the major allele frequencies in the two populations) fails to measure divergence when one of the populations has an allele frequency of 0.5 - substituting *p *= 0.5 in the formula above results in 0.5(1 *− q*) + (1 *− *0.5)*q*, and it is easy to see that nucleotide diversity will be 0.5 regardless of *q*.

Absolute difference in allele frequencies (*|p − q|*) is perhaps better, but consider populations where one allele's frequencies in the two populations are 0.4 and 0.6, respectively. Assigning an individual to a population based on observing this allele not inspire a lot of confidence in the result, they are roughly equally likely. Although the difference between allele frequency is the same for a site with allele frequencies 0.05 and 0.25, observing this allele is here five times as likely in one population as in the other, intuitively making this a much more useful site to observe.

For diagnostic SNP, what we really would like to know is the amount of *information *observing each site contributes. Using Bayes theorem, observing an allele *a *in some individual *N*, gives us the following posterior probability for *N *belonging to some population *A*, where the allele frequency, *P*(*a|A*), is known:

$$P\left(A|a\right)=\frac{P\left(a|A\right)}{P\left(a\right)}P\left(A\right)$$

Here, P(A) is our prior probability of *N *belonging to *A*, which after observing *a *is modified by a factor of

$$\frac{P\left(a|A\right)}{P\left(a\right)}$$

In order to assign *N *to one of several populations (either *A *or *B*, say), we are interested in the *relative *probabilities for the two hypotheses. In other words, we would like to know the *odds *for *N *belonging to one population or the other. Given the probabilities of *P*(*a|A*) and *P*(*a|B*), and initial odds *P *(*A*)/*P*(*B*), we get

$$\frac{P\left(A|a\right)}{P\left(B|a\right)}=\frac{P\left(a|A\right)P\left(A\right)/P\left(a\right)}{P\left(a|B\right)P\left(B\right)/P\left(a\right)}$$

Canceling out *P*(*a*), we find that the prior odds are modified by:

$$\frac{P\left(a|A\right)}{P\left(a|B\right)}$$

That is, the ratio of this allele's frequencies in each of the populations. For practical reasons, we take the logarithm of the odds. This gives us scores that are additive and symmetric (so that switching the two populations gives us the same score with the opposite sign). Specifically, base two logarithms will give us the score in bits.

When observing a site, we may of course also encounter the alternative allele. By the same reasoning as above, we find that this allele modifies the prior odds by

$$\frac{1-P\left(a|A\right)}{1-P\left(a|B\right)}$$

Lacking any specific information about priors, we can consider each population equally likely, and the likelihood of observing a particular allele is the average of the likelihood in each population. The information gain from each possible allele is then averaged, weighted by this average likelihood. For a biallelic site with major allele frequencies *p *and *q *(and consequentially, minor allele frequencies of 1 *− p *and 1 *− q*) in the two populations, the expected added information from the site then becomes:

$$I\left(p,q\right)=\frac{p+q}{2}\;\left|{\text{log}}_{2}\frac{p}{q}\right|+\left(1-\frac{p+q}{2}\right)\;\left|{\text{log}}_{2}\frac{1-p}{1-q}\right|$$

Note that we are here only interested in the amount of information gained, regardless of which hypothesis it favors, and thus we take the absolute values. For a site with multiple alleles enumerated by *i *and with frequency vectors **p **and **q **in the two populations, this generalizes to:

$$I\left(p,\;q\right)=\underset{i}{\sum }\frac{p_{i}+q_{i}}{2}\;\left|{\text{log}}_{2}\frac{p_{i}}{q_{i}}\right|$$

Returning to the example at the start of the section, we now find that a site with allele frequencies of 0.4 and 0.6 contributes 0.58 bits of expected information, while 0.05 and 0.25 contributes 2.32 bits. Unlike measures like *F_ST_*, measures of *I *is *additive *(assuming independence between sites), so the information gained from observing multiple sites is readily calculated, and observing with an ESI of 2.32 bits is equivalent to observing four sites with ESI 0.58.

It may also be instructive to compare this procedure to sequence alignment and position specific score matrices (PSSMs). In sequence alignment, a sequence of nucleotides or amino acids are scored by comparing its match to a target sequence to its match to some base model using log odds scores. The base model to compare against is often implicit (typically using sequences of random composition), but more elaborate models is also possible ([14]). Similarly, position specific frequency matrices are often converted to position specific score matrices using log odds. Calculating the information value from a set of observed alleles is then analogous to scoring an "alignment" of the set of observed alleles to two different sets of allele frequencies.

### Allele frequency confidence intervals

In order to apply the above method in practice, we need to measure the allele frequencies in the population. This is problematic for two reasons: First, we do not have precise knowledge of the allele frequencies, we can only estimate them from our sample, which introduces a sampling bias. Second, the sequencing process introduces additional artifacts that add nose and bias to the data. For instance, sequencing errors often result in substitutions, which are observed as apparent alleles. In addition, sequences can be incorrectly mapped, contain contamination, the reference genome can contain collapsed repeats, and the chemistry of the sequencing process is usually also biased - for instance, coverage is often biased by GC content. These artifacts often give the false appearance of variant positions.

One challenge with calculating site information from sequencing data (as opposed to using allele frequencies directly), is that such errors in the data can vastly overestimate the information content. For instance, an allele that appears to be fixed in one population means that any other observed allele will assign the individual to the alternative population - regardless of any other alleles. It is easy to see that an allele frequency of zero results in the odds going either to zero or infinity, and thus the log odds will go to either positive or negative infinity.

For diagnostic SNP discovery, it is more important to ensure that identified SNPs are informative, than to precisely estimate the information content. Thus, we take a conservative approach and use upper and lower limits for the allele frequencies by calculating confidence intervals using the method by Agresti and Coull [15]. In addition, the limits are also adjusted by a factor *∈*, corresponding to sequencing error rate. In the following, we will refer to the resulting measure as *conservative *site information, or CSI.

## Results

### Simulated data

A set of simulated reads were generated using FlowSim [16], using a procedure adapted to populations genetics studies [17]. A section of 10 megabases, comprised of the four largest scaffolds, was extracted from the salmon louse (*Lepeophtheirus salmonis*) draft genome assembly. Random substitutions were introduced at a rate of 1/200 bases to generate three different haplotypes, which where then admixed in proportions 1:2:3 and 3:2:1 to generate two population, *P*_1 _and *P*_2 _with variant allele frequencies of 0.17, 0.33 and 0.50, as shown in Table 1.

**Table 1:** Statistics for the variants from haplotypes H1, H2, and H3, as mixed in populations *P*^1 ^and *P*^2^.

haplotype	MAF, *P*_1_	MAF, *P*_2_	*F_ST_*	ESI
H1	0.17	0.50	0.125	1.6
H2	0.33	0.33	0	0
H3	0.50	0.17	0.125	1.6

Simulated reads were then generated with genome coverages of 10x, 20x, and 40x from each of the populations, using substitution rates of 0.002, 0.01 and 0.02. To simplify analysis, the indel rate was held constant at 0.001.

The reads were mapped to the reference genome using the BWA short read mapper [18], and analyzed using Samtools' mpileup command [19]. In addition to the methods described here, Popoolation [20] was used to calculate *F_ST _*and *p*-values from Fisher's exact test.

#### CSI scores for divergent and non-divergent sites

**Figure 1:**
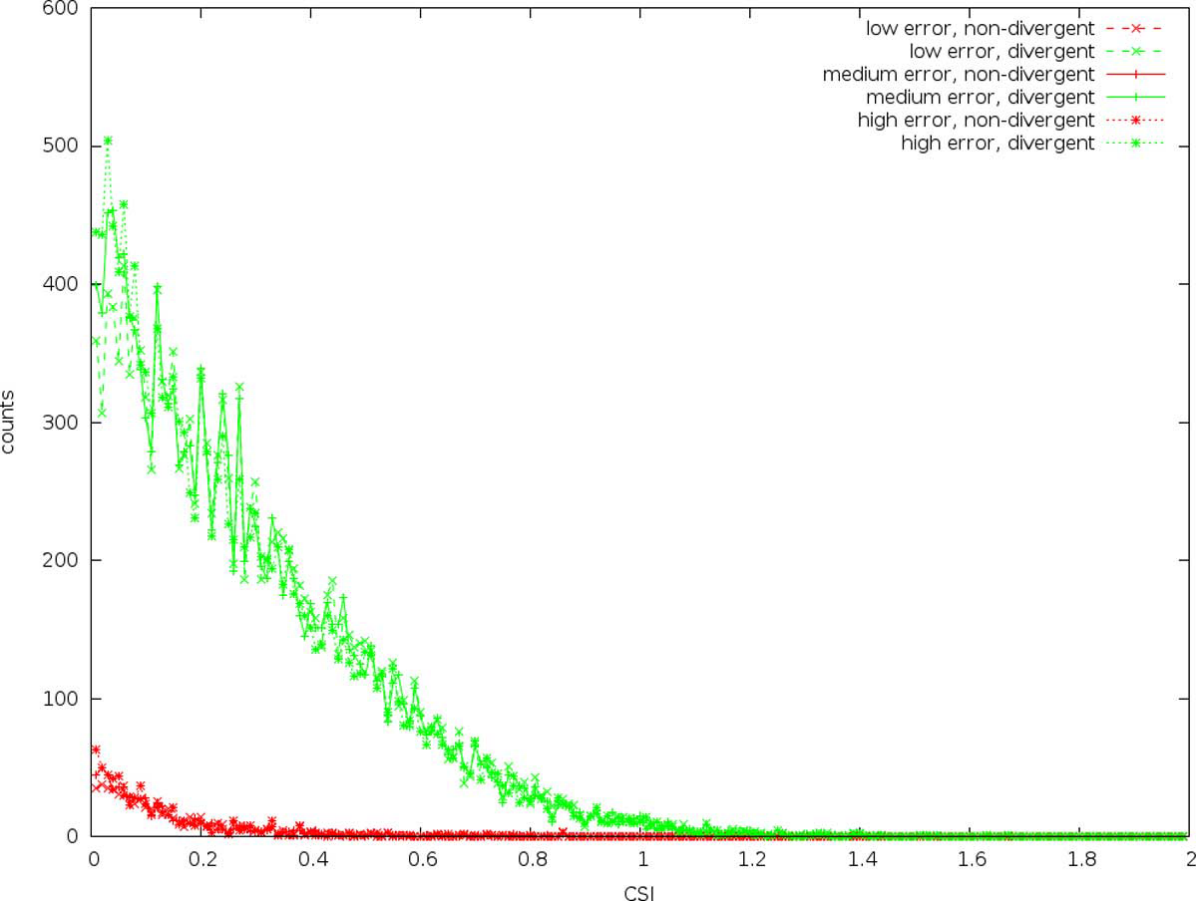
Expected site information (CSI) calculated on divergent and non-divergent sites in simulated reads with an expected coverage of 20x and error rates of 0.002, 0.01, and 0.02.

In Figure 1 we see that CSI clearly separates the divergent and non-divergent sites. At low CSI scores, the separation is approximately a factor of 10, and it increases with increasing CSI scores to a factor of about 100. Here, the number of divergent and non-divergent sites are approximately equal. The actual CSI value for the divergent sites are 1.6, we see that the program provides a conservative estimate, and only three sites score higher. The error rate does not seem to affect scores to a large degree.

**Figure 2:**
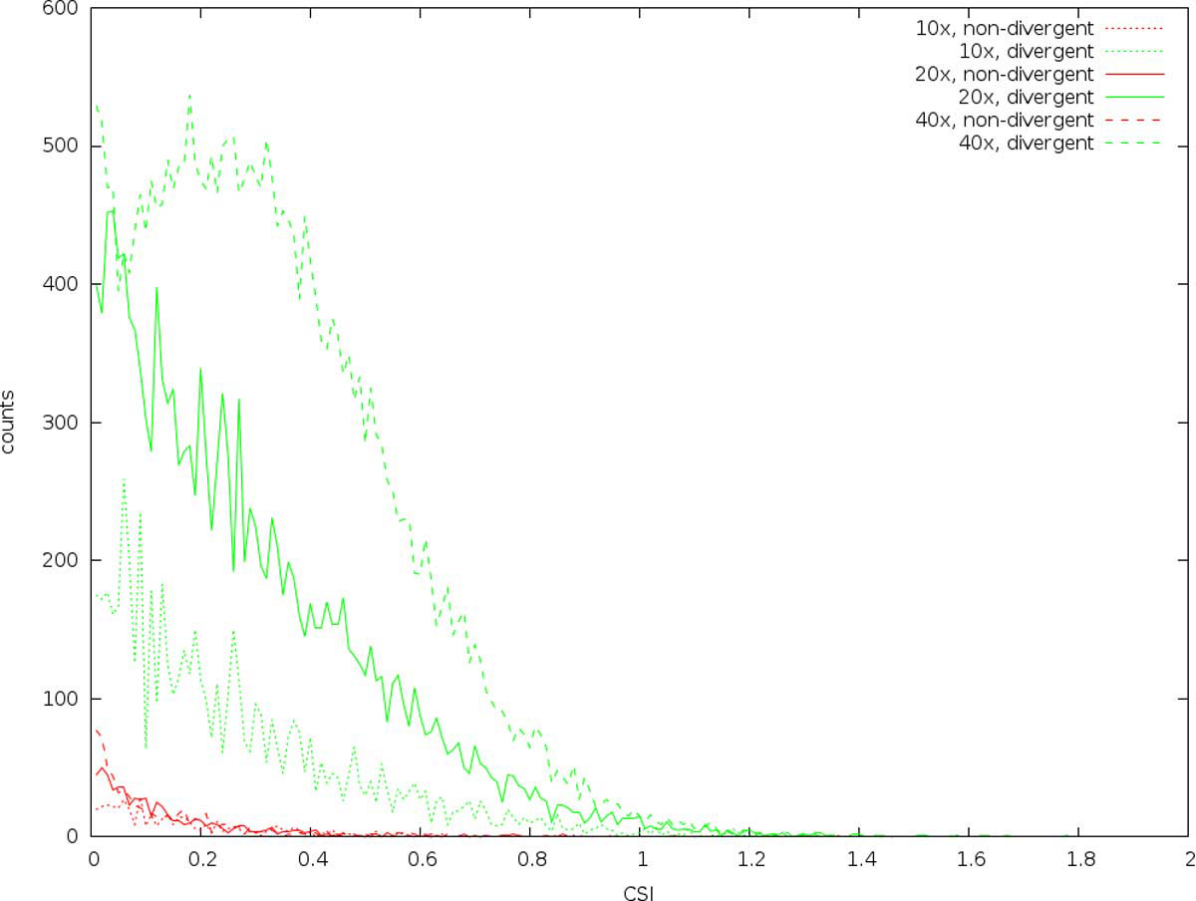
**Expected site information (CSI) calculated on divergent and non-divergent sites in simulated reads using various coverages**. The error rate is 0.01.

Figure 2 shows how the scores are affected by varying the coverage. Although false positive scores aren't markedly affected by variations in coverage, the scores for divergent sites increase substantially as coverage increases. This indicates that, at least for the coverages and error rates studied here, controlling variation in coverage (and sampling bias) is more important than substitution errors.

#### Comparing CSI and traditional statistics

**Figure 3:**
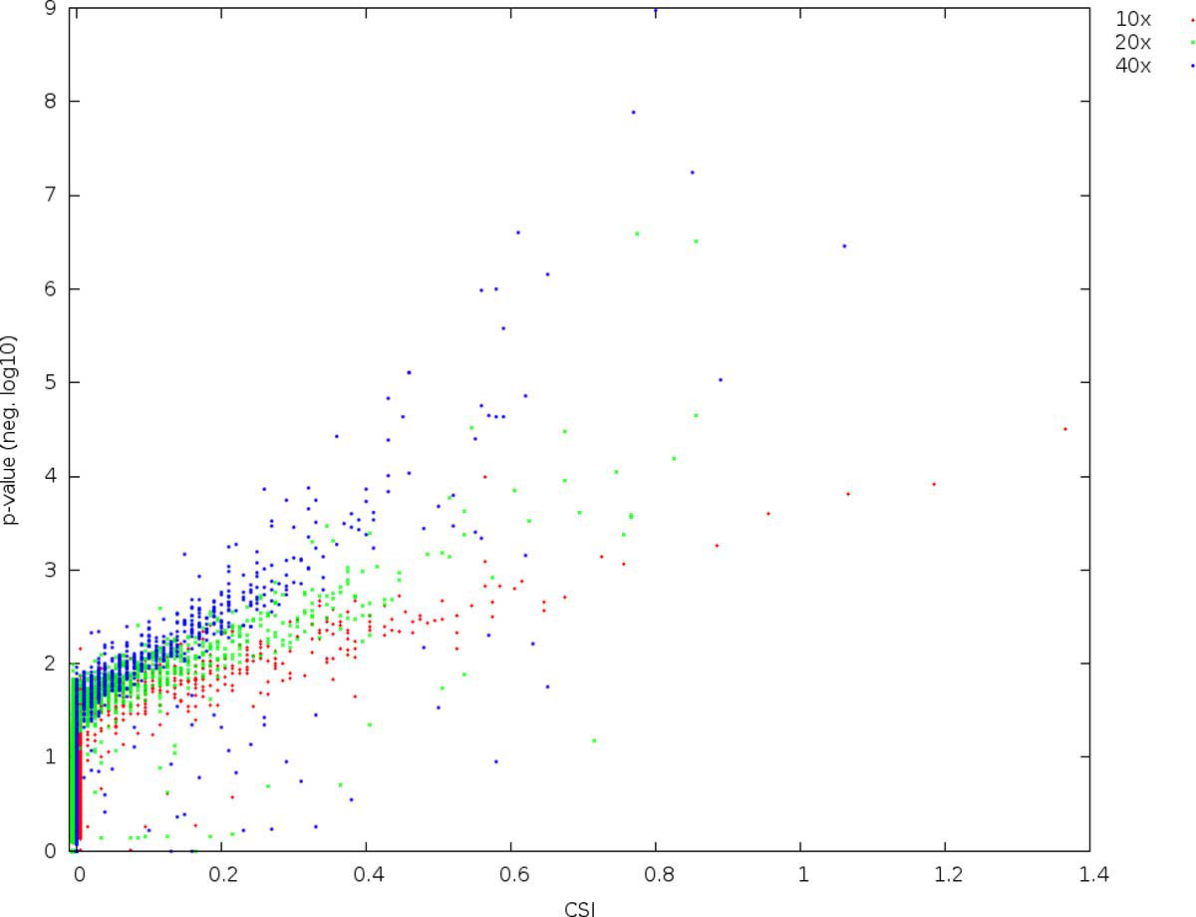
**Comparing the expected site information (CSI) with *p*-values using Fisher's exact test at various coverages and with a substitution rate of 0.01**. All sites are non-divergent.

The relationship between CSI and *p*-values is shown in Figure 3. Although all sites are non-divergent, we obtain many higher *p*-values at higher coverage, indicating that Fisher's exact test is upwardly biased as coverage increases.

**Figure 4:**
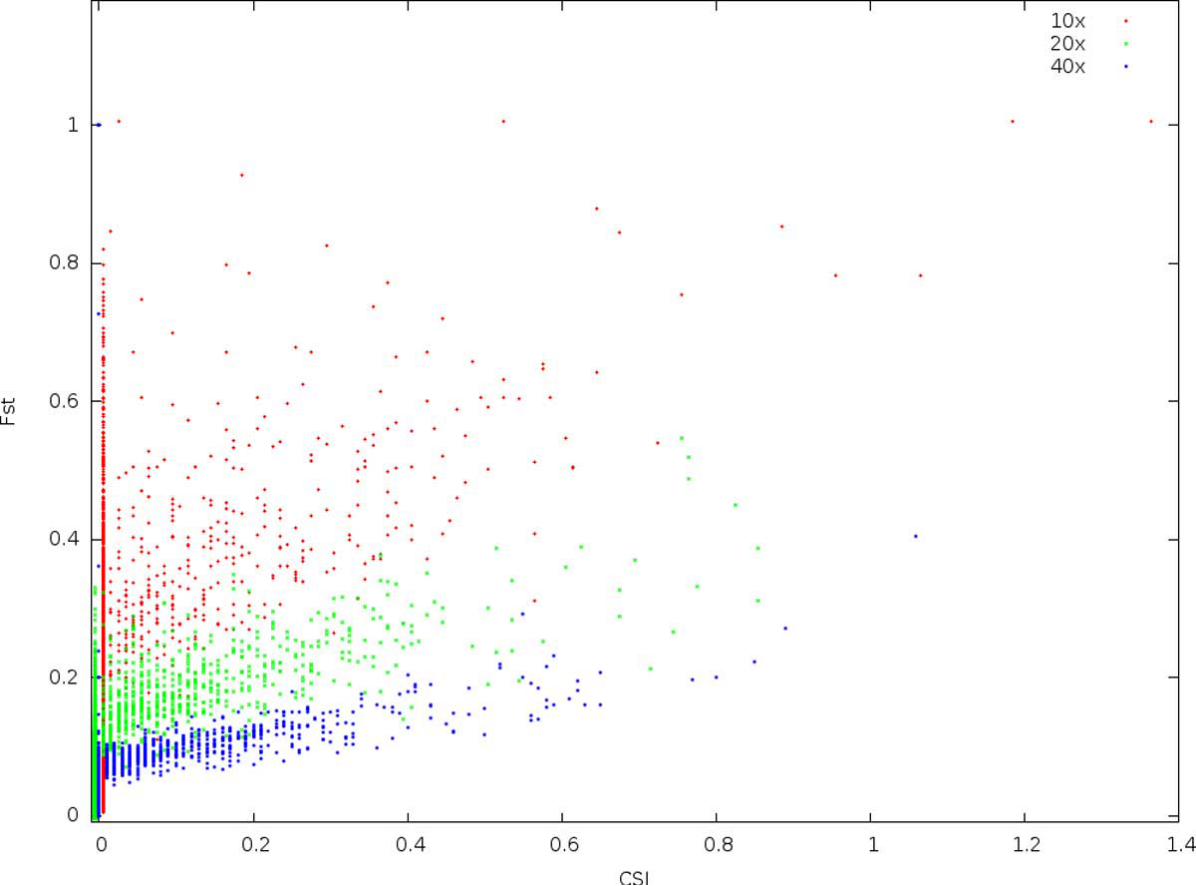
**Comparing the expected site information (CSI) with *F_ST _*values at various coverages and with a substitution rate of 0.01**. All sites are non-divergent, i.e. the real *F_ST _*and CSI are both 0.

In Figure 4, the relationship between CSI and *F_ST _*is explored. Especially, at low coverage, many sites show a very high *F_ST _*value. As coverage increases, both the variation and expectation of *F_ST _*is reduced, and also the correlation between CSI and *F_ST _*improves.

**Figure 5:**
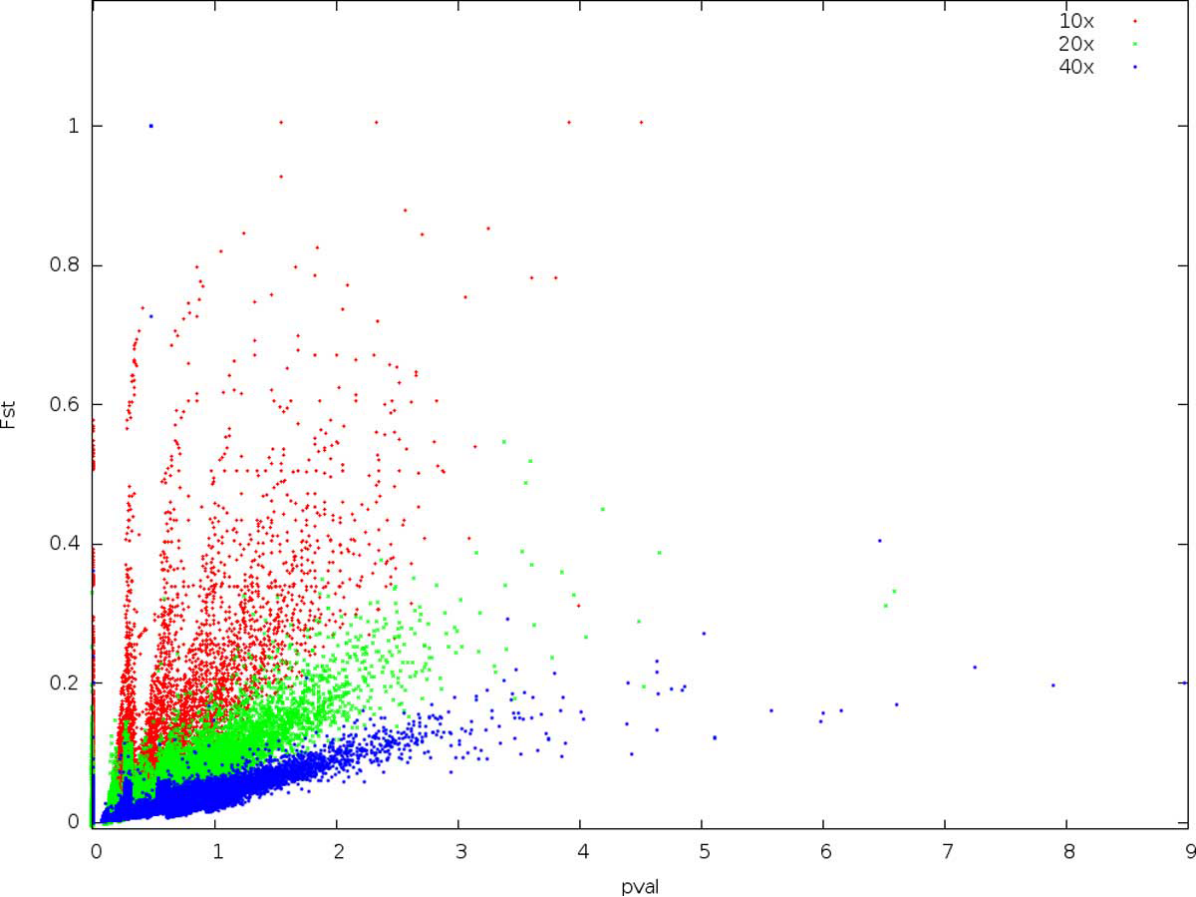
**Comparing *p*-value with *F_ST _*values for non-divergent sites at various coverages and with a substitution rate of 0.01**.

It is also instructive to contrast *F_ST _*values with *p*-values (Figure 5). Since most polymorphic sites result in a non-zero *p*-values and *F_ST_*, the diagram is noticeably denser than Figures 3 or 4. There is also a clear banding effect; as coverage increases, *F_ST _*values tend to decrease, and *p*-values increase, and the bulk of the data is rotated in a clockwise direction.

#### Comparing CSI and F_ST _for divergent and non-divergent sites

**Figure 6:**
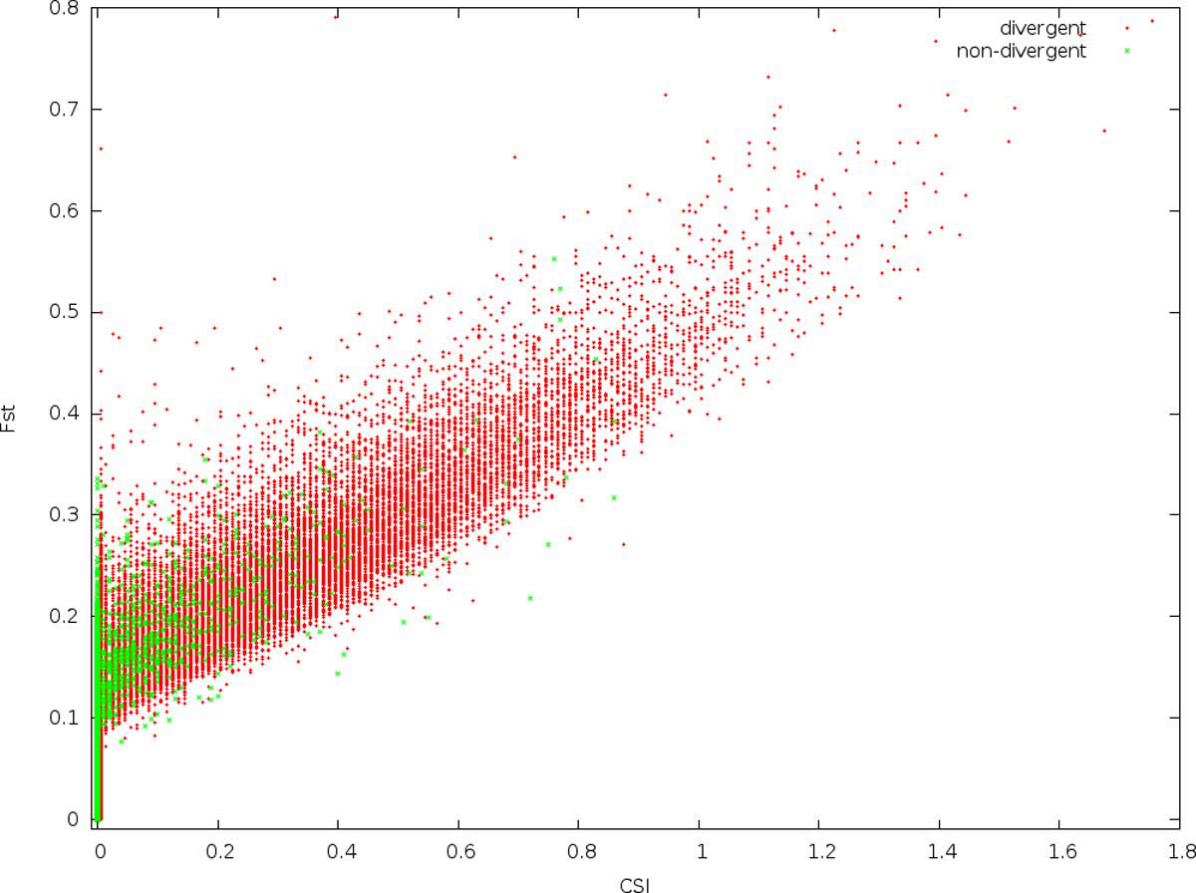
**Comparing the expected site information (CSI) with *F_ST _*for divergent and non-divergent sites, using a 20x coverage and a substitution rate of 0.01**.

Adding the divergent sites in Figure 6, we see that the distribution for the divergent sites extend the general distribution for the non-divergent sites.

### Real data

One important use for SNPs, is to assign individuals to their respective populations or subpopulations. For instance, the quantity of Norwegian farmed salmon exceeds the wild river populations by a large factor. As salmon occasionally escape from sea farms, the ability to effectively identify escapees is important both to identify the farms responsible, as well as quantifying the ecological effects of introgression. Here SNPs will play an important role by providing a low-cost, high resolution data [21].

Below, we examine pooled salmon sequencing data from rivers Flekkeelva and Suldalselva, and investigate the resulting CSI distributions. From each of the rivers, two pools were sequenced using Illumina HiSeq, resulting in datasets *F*_1_, *F*_2_, *S*_1 _and *S*_2_, each containing between 346 and 397 million aligned reads, corresponding to coverages of 11.5x to 13.2x, assuming a genome size of 3 gigabases. The data sets were then merged by river (combining *F*_1 _with *F*_2 _and *S*_1 _with *S*_2_), and by replicate (combining *F*_1 _with *S*_1 _and *F*_2 _with *S*_2_, to provide a model for false discoveries).

#### Comparison

**Figure 7:**
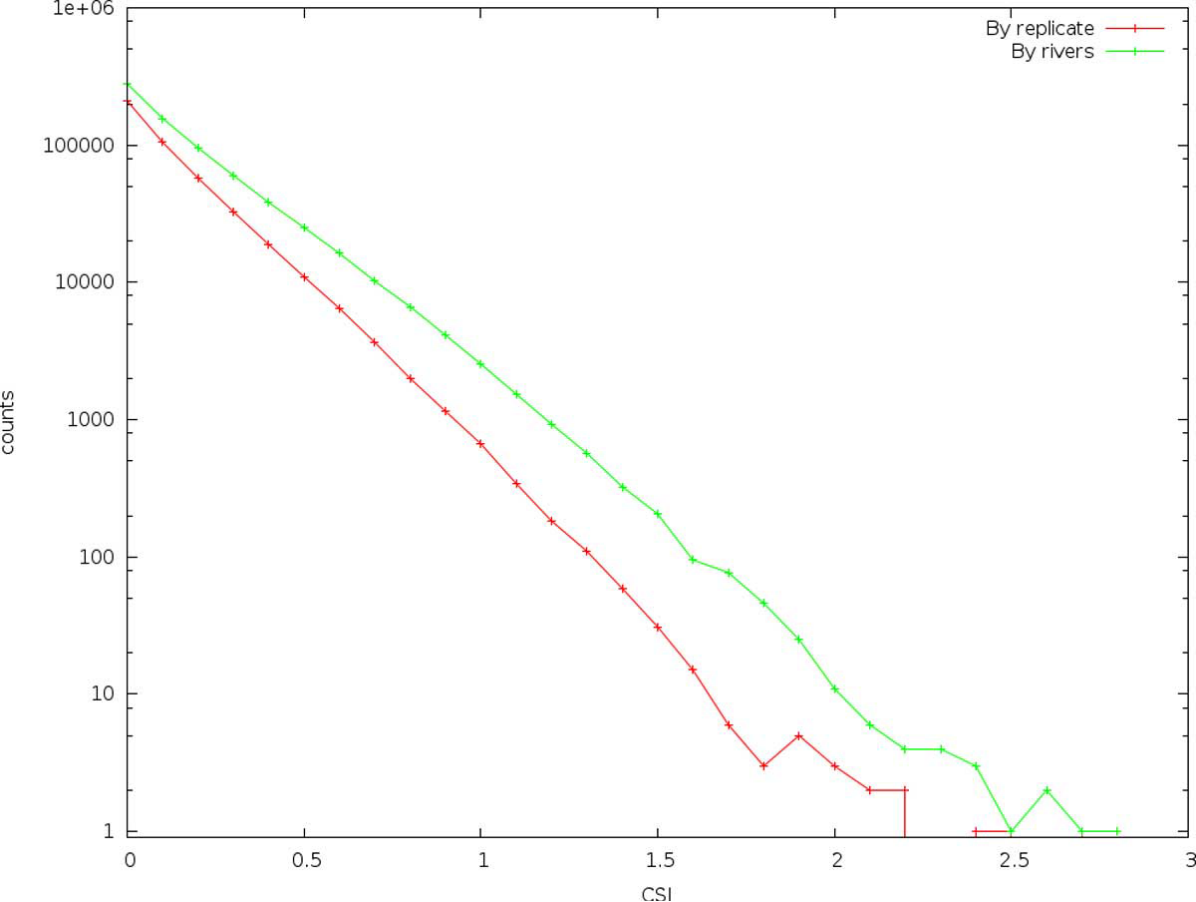
**Comparing the expected site information (CSI) between pools mixed from both rivers, and single-river pools**.

It can be seen from Figure 7 that, as expected, we identify a larger number of sites when comparing between rivers than between the mixed replicates, and although the separation is not as clear as for the simulated data, the slope is similar. The difference increases (and thus identification accuracy) with increasing scores, but there remain several high-scoring sites also in the replicates comparison.

#### Filtering by coverage

**Figure 8:**
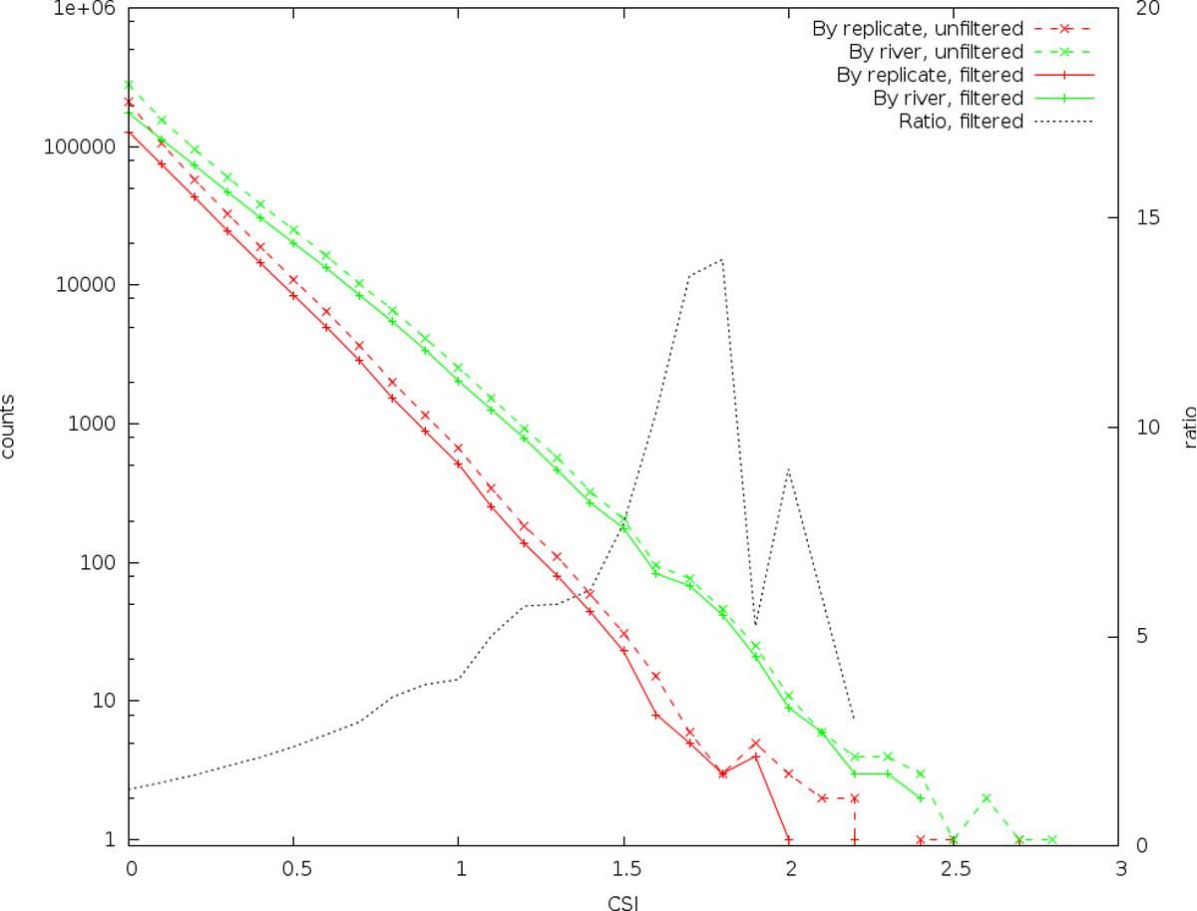
**Comparing the expected site information (CSI) between pools mixed from both rivers, and single-river pools, filtered by coverage**. The right axis shows the ratio of between-river scores to between-replicates scores for the coverage-filtered sites.

A closer examination of the data reveals that many of the high scoring sites have a coverage substantially higher than the expected combined coverage of approximately 50x. This can be due to collapsed repeats in the genome assembly or other artifacts of the assembly, sequencing, or mapping processes. To investigate this, sites were filtered by coverage, retaining only sites with a total coverage of 50 ± 20 (which corresponds to roughly three standard deviations of a Poisson distribution). The results are shown in Figure 8, for comparison the unfiltered results from Figure 7 are retained with dashed lines.

We see from Figure 8 that filtering on coverage eliminates some of the noise, most noticeable for higher CSI values. The effect of filtering also tends to reduce the scores between the replicate pools more than the river pools, this observation is also supported by the total number of identified SNP, as summarized in Table 2. In all cases a large number of sites are removed, 30% (rivers) and 33% (replicates) of the identified sites.

**Table 2:** The number of sites with non-zero expected site information, both in absolute numbers and in percent of an estimated 3 gigabase genome, before and after filtering for coverage.

Comparison	# sites	% genome
Between rivers	699970	0.023
Between rivers, filtered	493163	0.016
Between replicates	450814	0.015
Between replicates, filtered	303972	0.010

## Discussion

### Statistics, coverage, and sequencing errors

It is striking that *p*-values for the non-divergent sites increase with coverage. For instance, out of the 36000 non-divergent sites, we expect approximately 36 sites by chance to have a *p*-value less than 10^−3^. For 10x coverage, we find 9, for 20x, we find 35, and for 40x we find 70. This indicates that *p*-values are biased upwards with increasing coverage, and must be consequently be interpreted with care [13]. The expectation and variance of *F_ST _*similarly depends on coverage. In contrast, low coverage in combination with sequencing errors and incorrectly mapped reads here result in a large number of high-scoring non-divergent sites. Using a combination of these measures may be effective, but also effectively narrows the data set, much like a stringent filtering for coverage.

Simulated data is by definition a simplification of reality. For instance, here the data assumes uniform probability of reads across the genome, and unbiased and context independent sequencing errors. Also, divergent and non-divergent positions occur in similar numbers in the simulated data, in reality, there will be a continuous spectrum of allele frequencies, and it will depend globally on the degree of divergence between populations, and locally on selection and other non-random evolution pressures. Results from simulated data must, as always, be interpreted as optimistic. In practice, coverage will vary substantially across a sequenced genome. In general, high variance regions tend to have lower mapping [22], but other factors are bias caused by GC-content, misassembly and collapsed repeats, copy-number and other structural variations, incorrect mapping, sampling bias (including from variation of molarity in DNA samples). Real data sets must therefore be expected to contain a wide range of coverages, mapping reliability, and sequencing error rates.

### Other information theoretic measures

Although not commonly applied, information theoretic measures have been used previously in analyzing genetic variation. Expected site information is related to Kullback-Leibler divergence [23], but differs in that it is symmetric and extended to multiple alleles. Rosenberg [6] gives a summary of several alternative statistics, and also develops an information theoretic measure that contrasts individual populations with an average of all population. This measure is then used to infer ancestry, and applied to microsatellite data. Here, we develop an information theoretic measure in a Bayesian context, and apply it to high-throughput sequencing data.

### Dealing with sequencing errors and artifacts

Based on the assumption that most sequencing errors will be singletons, Achaz [24] developed variants of several estimators for Θ which avoids taking singletons into account. Achaz' formulas were later adapted to high-throughput sequencing experiments, and given a more generalized (but approximate) form that allowed an arbitrary lower bound on number of observed alleles [4]. However, much of the genetic diversity is in the form of low frequency alleles, and as singletons also have a high impact on many statistics [24], these estimators have lower power [24,2]. It is also possible to attempt to quantify the errors more precisely by leveraging characteristics of the data [5].

### Future work

Here, we have focused on the *expected *information content. As this is an additive measure, it is straightforward to sum over multiple sites to get the expected information for a set of SNPs. Since rare alleles yield more information than common ones, a natural extension might be to consider instead the *minimum *information content from a set of loci, ensuring that we can reach a conclusion even if we are unlucky with the actual alleles observed. Yet another option is to calculate a confidence interval for the information.

## Conclusions

When selecting diagnostic SNPs, we want to find sites that provide the most information regarding our current problem. Although this is commonly measured using statistics like *F_ST_*, these are *indirect *measurements, proxies for the actual information. In addition, we have seen that it and other commonly used statistics have intrinsic biases when applied to sequencing data, due to coverage variation, sequencing artifacts, and mapping errors.

As an alternative, we have derived a direct calculation of the expected site information from allele frequencies, using a Bayesian framework. In addition to being a direct measurement of the value of interest, it has a clear interpretation, and desirable properties, like additivity. We have further developed a conservative estimator for this statistic, and provide an implementation.

## Availability

The method as described above was implemented in a program, 'varan', which parses read alignments in the standard "mpileup" format as output by the samtools mpileup command. It can currently output several different statistics and estimators, including conservative expected site information (CSI). The software is distributed under the General Public License, and the source code can be downloaded from http://malde.org/~ketil/biohaskell/varan. Further information and documentation is available from http://biohaskell.org/Applications/Varan.

Simulation data, tables, and scripts used in this paper is available from http://malde.org/~ketil/papers/varan. The salmon louse genome used to generate the simulated reads is available from http://sealouse.imr.no/.

## Competing interests

The author declares that they have no competing interests.

## Declarations

Publication costs for this article were funded by the Institute of Marine Research.

This article has been published as part of *BMC Genomics *Volume 15 Supplement 6, 2014: Proceedings of the Twelfth Annual Research in Computational Molecular Biology (RECOMB) Satellite Workshop on Comparative Genomics. The full contents of the supplement are available online at http://www.biomedcentral.com/bmcgenomics/supplements/15/S6.

## References

[B1] Collins FS Brooks LD Chakravarti A A DNA polymorphism discovery resource for research on human genetic variation Genome research 1998 8 12 1229 1231 9872978 10.1101/gr.8.12.1229

[B2] Cutler DJ Jensen JD To pool, or not to pool? Genetics 2010 186 1 41 43 10.1534/genetics.110.121012 20855575 PMC2940305

[B3] Altmann A Weber P Quast C Rex-Haffner M Binder EB Müller-Myhsok B vipR: variant identification in pooled DNA using R Bioinformatics [ISMB/ECCB] 2011 27 13 77 84 10.1093/bioinformatics/btr205 PMC3117388 21685105

[B4] Futschik A Schlötterer C The next generation of molecular markers from massively parallel sequencing of pooled DNA samples Genetics 2010 186 1 207 218 10.1534/genetics.110.114397 20457880 PMC2940288

[B5] Bansal V Harismendy O Tewhey R Murray SS Schork NJ Topol EJ Frazer KA Accurate detection and genotyping of SNPs utilizing population sequencing data Genome research 2010 20 4 537 545 10.1101/gr.100040.109 20150320 PMC2847757

[B6] Rosenberg NA Li LM Ward R Pritchard JK Informativeness of genetic markers for inference of ancestry The American Journal of Human Genetics 2003 73 6 1402 1422 10.1086/380416 PMC1180403 14631557

[B7] Zhou N Wang L Effective selection of informative SNPs and classification on the hapmap genotype data BMC Bioinformatics 2007 8 1 484 10.1186/1471-2105-8-484 18093342 PMC2245981

[B8] Fumagalli M Vieira FG Korneliussen TS Linderoth T Huerta-Sánchez E Albrechtsen A Nielsen R Quantifying population genetic differentiation from next-generation sequencing data Genetics 2013 195 3 979 992 10.1534/genetics.113.154740 23979584 PMC3813878

[B9] Weir BS Hill W Estimating F-statistics Annual Review of Genetics 2002 36 1 721 750 10.1146/annurev.genet.36.050802.093940 12359738

[B10] Holsinger KE Weir BS Genetics in geographically structured populations: defining, estimating and interpreting *F_ST_* Nature Reviews Genetics 2009 10 9 639 650 10.1038/nrg2611 19687804 PMC4687486

[B11] Karlsson EK Baranowska I Wade CM Hillbertz NHS Zody MC Anderson N Biagi TM Patterson N Pielberg GR Kulbokas EJ Efficient mapping of mendelian traits in dogs through genome-wide association Nature genetics 2007 39 11 1321 1328 10.1038/ng.2007.10 17906626

[B12] Jost L GST and its relatives do not measure differentiation Molecular Ecology 2008 17 18 4015 4026 10.1111/j.1365-294X.2008.03887.x 19238703

[B13] Lin M Lucas HC Jr Shmueli G Research commentary-too big to fail: Large samples and the p-value problem Information Systems Research 2013 24 4 906 917 10.1287/isre.2013.0480

[B14] Malde K The effect of sequence quality on sequence alignment Bioinformatics 2008 24 7 897 900 10.1093/bioinformatics/btn052 18296747

[B15] Agresti A Coull BA Approximate is better than "exact" for interval estimation of binomial proportions The American Statistician 1998 52 2 119 126

[B16] Balzer S Malde K Lanzén A Sharma A Jonassen I Characteristics of 454 pyrosequencing data--enabling realistic simulation with FlowSim Bioinformatics 2010 26 18 420 425 10.1093/bioinformatics/btq365 PMC2935434 20823302

[B17] Malde K Simulating a population genomics data set using FlowSim BMC Research Notes 2014 7 1 68 10.1186/1756-0500-7-68 24479665 PMC3942619

[B18] Li H Durbin R Fast and accurate short read alignment with burrows-wheeler transform Bioinformatics 2009 25 14 1754 1760 10.1093/bioinformatics/btp324 19451168 PMC2705234

[B19] Li H Handsaker B Wysoker A Fennell T Ruan J Homer N Marth G Abecasis G Durbin R The sequence alignment/map format and samtools Bioinformatics 2009 25 16 2078 2079 10.1093/bioinformatics/btp352 19505943 PMC2723002

[B20] Kofler R Orozco-terWengel P De Maio N Pandey RV Nolte V Futschik A Kosiol C Schlötterer C Popoolation: a toolbox for population genetic analysis of next generation sequencing data from pooled individuals PLoS One 2011 6 1 15925 10.1371/journal.pone.0015925 PMC3017084 21253599

[B21] Karlsson S Moen T Lien S Glover KA Hindar K Generic genetic differences between farmed and wild atlantic salmon identified from a 7K SNP-chip Molecular Ecology Resources 2011 11 s1 247 253 21429178 10.1111/j.1755-0998.2010.02959.x

[B22] Wang W Wei Z Lam TW Wang J Next generation sequencing has lower sequence coverage and poorer SNP-detection capability in the regulatory regions Scientific reports 2011 1 55 10.1038/srep00055 22355574 PMC3216542

[B23] Kullback S Leibler RA On information and sufficiency The Annals of Mathematical Statistics 1951 79 86

[B24] Achaz G Testing for neutrality in samples with sequencing errors Genetics 2008 179 3 1409 1424 10.1534/genetics.107.082198 18562660 PMC2475743

